# c-Jun, Foxo3a, and c-Myc Transcription Factors are Key Regulators of ATP-Mediated Angiogenic Responses in Pulmonary Artery Vasa Vasorum Endothelial Cells †

**DOI:** 10.3390/cells9020416

**Published:** 2020-02-11

**Authors:** Derek Strassheim, Vijaya Karoor, Hala Nijmeh, Philip Weston, Martin Lapel, Jerome Schaack, Timothy Sullivan, Edward C. Dempsey, Kurt R. Stenmark, Evgenia Gerasimovskaya

**Affiliations:** 1 Department of Medicine Cardiovascular and Pulmonary Research Laboratory, University of Colorado Denver, Aurora, CO 80045, USA; derek.strassheim@cuanschutz.edu (D.S.); vijaya.karoor@cuanschutz.edu (V.K.); timothy.sullivan@cuanschutz.edu (T.S.); edward.dempsey@cuanschutz.edu (E.C.D.); kurt.stenmark@cuanschutz.edu (K.R.S.); 2 Department of Pediatrics, Division of Critical Care Medicine, University of Colorado Denver, Aurora, CO 80045, USA; hala.nijmeh@cuanschutz.edu (H.N.); philip.weston@compass.com (P.W.); lapel.martin@gmail.com (M.L.); 3 Department of Microbiology, University of Colorado Denver, Aurora, CO 80045, USA; jerry.schaack@cuanschutz.edu; 4 Rocky Mountain Regional VA Medical Center, Aurora, CO 80045, USA

**Keywords:** vasa vasorum, angiogenesis, endothelial cells, extracellular ATP, Akt, mTOR transcription factors, c-Jun, Foxo3a, c-Myc

## Abstract

Angiogenic vasa vasorum (VV) expansion plays an essential role in the pathogenesis of hypoxia-induced pulmonary hypertension (PH), a cardiovascular disease. We previously showed that extracellular ATP released under hypoxic conditions is an autocrine/paracrine, the angiogenic factor for pulmonary artery (PA) VV endothelial cells (VVECs), acting via P2Y purinergic receptors (P2YR) and the Phosphoinositide 3-kinase (PI3K)-Akt-Mammalian Target of Rapamycin (mTOR) signaling. To further elucidate the molecular mechanisms of ATP-mediated VV angiogenesis, we determined the profile of ATP-inducible transcription factors (TFs) in VVECs using a TranSignal protein/DNA array. C-Jun, c-Myc, and Foxo3 were found to be upregulated in most VVEC populations and formed nodes connecting several signaling networks. siRNA-mediated knockdown (KD) of these TFs revealed their critical role in ATP-induced VVEC angiogenic responses and the regulation of downstream targets involved in tissue remodeling, cell cycle control, expression of endothelial markers, cell adhesion, and junction proteins. Our results showed that c-Jun was required for the expression of ATP-stimulated angiogenic genes, c-Myc was repressive to anti-angiogenic genes, and Foxo3a predominantly controlled the expression of anti-apoptotic and junctional proteins. The findings from our study suggest that pharmacological targeting of the components of P2YR-PI3K-Akt-mTOR axis and specific TFs reduced ATP-mediated VVEC angiogenic response and may have a potential translational significance in attenuating pathological vascular remodeling.

## 1. Introduction

Pathologic vascular remodeling plays a critical role in the development of cardiovascular diseases, including pulmonary hypertension (PH). Accumulating evidence suggests that vasa vasorum (VV), a microcirculatory network that provides oxygen and nutrients to the adventitia and media of large blood vessels, is a key factor in the vascular remodeling process in both systemic and pulmonary circulation [1,2,3,4,5,6,7]. Our previous studies revealed a marked expansion of the VV network in the adventitia of the pulmonary artery (PA) of chronically hypoxic hypertensive calves—a process that was accompanied by adventitial thickening and infiltration of circulating inflammatory cells into the arterial wall, implicating the VV in pathologic vascular remodeling in PH [3,8,9,10]. Although it is likely important in pathological vascular remodeling, little is known about the molecular mechanisms underlying these pathological events.

Among hypoxia-inducible vasoactive mediators, such as growth factors, cytokines, and peptide hormones, extracellular purines (ATP, ADP, and adenosine) are critical signaling molecules regulating proliferative, contractile and inflammatory responses in the vasculature. Under pathological conditions, including hypoxia and inflammation, ATP is released from vascular and blood cells and mediates its effect by acting on P2Y (G protein-coupled) and P2X (metabotropic) purinergic receptors [11,12,13,14,15,16,17]. However, the role of extracellular purines and mechanisms of their action in VV pathological expansion remains largely unexplored. Our previous studies demonstrated that ATP could be released from PA adventitial fibroblasts and VV endothelial cells (VVECs) in response to hypoxia, and play an autocrine/paracrine role in mitogenic and angiogenic responses in VVECs via the activation of P2Y1 and P2Y13 purinergic receptors [13,18,19]. We also found that PI3K-Akt and Rho-ROCK pathways play a critical role in both hypoxia-induced ATP release and ATP-mediated angiogenic responses in VVECs [18]. These observations are consistent with the established role of PI3K-mTOR Akt-pathways in cell proliferation [20,21,22,23,24]. They also support the concept that sustained VVEC activation by autocrine-paracrine purinergic signaling in hypoxia may induce the formation of a structurally abnormal VV network that recapitulates the vascular aberrations seen with tumor- and inflammation-driven angiogenesis.

Endothelial gene expression coordinates different steps in angiogenesis, such as proteolysis of the extracellular matrix, destabilization of the cellular junction, and proliferation, migration, and differentiation of vascular cells [25,26,27,28,29,30,31]. Multiple transcription factors (TFs) acting in a combinatorial fashion, are involved in endothelial-specific regulation of genes associated with angiogenesis, tissue remodeling, and vessel stabilization [32]. Extracellular purines, the ligands for G protein-coupled P2Y receptors, regulate complex signaling networks, leading to the transcriptional control of gene expression [33]. However, activated TF profiles in angiogenic vessels, and in VV in particular, in response to purinergic stimulation remain unknown.

This study was undertaken to identify the ATP-inducible TFs in VVECs regulated by the PI3K-Akt-mTOR pathway and to examine the role of these TFs in VVEC angiogenesis. Using a TranSignal protein/DNA array and Ingenuity Pathway Analysis, we identified c-Jun, Foxo3a, and c-Myc as critical regulators of VVEC angiogenic responses. Furthermore, using siRNA-mediated knockdown (KD) of c-Jun, Foxo3a, and c-Myc, we identified target proteins involved in the regulation of the cell cycle, the extracellular matrix, adhesion, cell junction, and endothelial phenotype. Given that transcriptional mechanisms of VV angiogenic expansion remain under-investigated, our study provided, for the first time, evidence of the molecular mechanisms underlying this process. 

## 2. Materials and Methods

### 2.1. Cultures of VVEC

VVECs were purified from the PA adventitia of 14-day-old Holstein male calves that were exposed to hypobaric hypoxia for two weeks (P_B_ = 430 mmHg), as previously described [24]. Institutional guidelines were followed, and the procedure was approved by the Institutional Animal Care and Use Committee (Department of Physiology, School of Veterinary Medicine, Colorado State University, Ft. Collins, CO, USA). VVECs were isolated from the vascularized areas of PA adventitia and grown in Dulbecco’s Modified Eagle Medium (DMEM) supplemented with 10% fetal bovine serum (FBS) and endothelial growth supplement (Upstate Biotechnology, Charlottesville, VA, USA). VVECs have been shown to express endothelial markers, including vWF, eNOS, and PECAM-1, binding of the lectin *Lycopersicon Esculentum*, and to incorporate acetylated low-density lipoproteins labeled with 1,1’-dioctadecyl-3,3,3,3’-tetramethylindo-carbocyanine perchlorate (DiI-Ac-LDL). All studies were performed on cells from passages 3 to 7. Under these conditions, the cells sustained consistent functional, morphological, and phenotypic characteristics.

### 2.2. siRNA Transfection

siRNA to c-Jun and c-Myc were obtained from Santa Cruz Biotechnology Inc. (Dallas, TX, USA). siRNA to Foxo3a was synthesized by Integrated DNA Technology (IDT, Skokie, IL, USA) based on a reported sequence in the bovine cell genome. Three VVEC populations obtained from different animals were transfected with TF-specific siRNA (10 nM) containing three different pooled siRNA or universal siRNA (10 nM) (Sigma-Aldrich St. Louis, MO, USA), using Dharmafect Reagent® (Dharmacon, Denver, CO) as per manufacturer recommendations. Universal siRNA (scramble) was used as a control. Cell lysates prepared from siRNA-treated cells were analyzed by Western blot to determine the efficacy of knockdown 48 h after transfection. The siRNA-mediated knockdown was in the range 70–80%. Cells in two 100 mm dishes were transfected with TF-specific siRNA and used for thymidine incorporation, migration, tube formation measurements, and WB analysis of angiogenesis-associated target proteins. The values of siRNA-treated cells were compared to scramble siRNA-treated cells from 3 different cell populations. 

### 2.3. Preparation of Nuclear Fraction 

Growth-arrested VVECs (72 h, DMEM without serum) were stimulated with ATP (100 μM) for 1, 6, and 12 h. At the end of the incubation, the cells were washed with ice-cold PBS, scraped off the dishes, and collected by centrifugation at 500 x g for 3 min at 4 °C. Nuclear extracts were prepared using NE-PER^TM^ kit (Piers, Rockford, IL, USA). Nuclear fractions were snap-frozen in liquid nitrogen in aliquots and stored at –80 °C. Protein concentration was determined by the Bradford method using the Bio-Rad protein assay kit with bovine serum albumin as a standard.

### 2.4. Analysis of Transcription Factor Transactivation

Twenty micrograms of nuclear protein were processed according to TranSignal Protein/DNA Array I and Array II kits (Panomics, Inc., Redwood City, CA, USA) according to the manufacturer’s instructions, without modifications. The National Institute of Health ImageJ program was used to determine the density of spots on the array. Data obtained were further analyzed using Morpheus (www.broadinstitute.org) and Ingenuity Pathway Analysis Software (IPA Qiagen 2.2). 

### 2.5. DNA Synthesis 

The cells were plated in 24-well plates in triplicates at a density of 1.2 × 10^4^ cells per well in DMEM supplemented with 10% FBS. After 72 h of growth arrest (DMEM without serum), the cells were pre-incubated with the PI3K/mTOR inhibitor, PI-103 (0.001-1 μM, 45 min), or remained untreated and were stimulated with extracellular (ATP 100 μM) in the presence of 0.125 μM Ci of [methyl-^3^H] thymidine (NEN Life Science Products, Boston, MA, USA) for 24 h. Incorporated radioactivity was determined as previously described [18]. 

### 2.6. Migration Assay 

Growth-arrested VVECs (1.0 × 10^5^ cells/well) were plated in 200 μl of serum-free DMEM in permeable cell culture inserts (8.0 μ pore size, Costar Inc, Milpitas, CA, USA), and precoated with 0.1% gelatin (Sigma, St. Louis, MO). Cells in transwells were pre-incubated with PI-103 (0.01, 0.1 and 1 μM) for 45 min or remained untreated. ATP (100 μM) was added to the lower chamber containing 800 μl of serum-free DMEM. After 24 h incubation, the cells remaining on the upper surface of the filter were wiped off and the migrated cells were fixed with methanol for 15 min and stained with 0.2% crystal violet in 2% (vol/vol) ethanol for 15 min. Images of the cells that migrated through the filter were obtained using a phase contrast microscope, at x40 magnification, from three random fields. 

### 2.7. Tube Formation Assay

Tube formation assay was carried out in ibidi angiogenesis slides (ibidi) precoated with 10 μl of Growth Factor Reduced Matrigel Matrix (BD Bioscience). Growth-arrested VVECs were seeded on polymerized Matrigel in triplicate at a density of 1.7 × 10^4^ cells/well. Cells were pre-incubated with PI-103 (0.5 μM, 45 min) or remained untreated and stimulated with 100 μM ATP for 8–10 h. Photographs were taken using a digital camera connected to a phase-contrast microscope (Nikon), at three to five random fields for each condition, at ×10 magnification. The average tube length and the number of tubes were quantified using ImageJ64 software. 

### 2.8. Western Blot Analysis 

Growth-arrested cells (72 h, DMEM without serum) were pre-incubated with PI-103 (100 nM) μM) for 45 min and stimulated with ATP (100 μM) for 10, 30, and 60 min. Total cell lysates were prepared as previously described [24]. Equivalent amounts of total cell protein (20–40 μg) were separated using sodium dodecyl sulfate-polyacrylamide gel electrophoresis (SDS-PAGE). Proteins were transferred to PVDF membranes and probed with antibodies against specific proteins. The antibodies were validated and provided by the established vendors (Appendix, Table A1). Immunoreactive bands were detected by ECL kit (Renaissance, NEN Life Science Product, Boston, MA, USA). Densitometry of the scanned images was quantitated using ImageJ64 software. Lamin A/C, GAPDH, or non-phosphorylated forms of protein kinases were used as loading controls, as specified in the figure legends. A Bio-Rad gel scanner and densitometer (Gel DocXR with Quantity 1 program) were used to assess the intensity of the bands obtained by Western blots. The pixel density for each of the proteins was normalized to the loading control and presented as fold change compared to the basal conditions. 

### 2.9. Statistical Analysis 

Data were analyzed using GraphPad Prism 4 (GraphPad Software for Science Inc., San Diego, CA). The results are presented as mean ± SEM. The significance of difference between two measurements in the Western blot analysis was determined by unpaired, two-tailed *t* tests. One-way analysis of variance was used for multiple comparisons followed by the Bonferroni post-test for evaluating the significance of the thymidine incorporation, migration, and tube formation assays. P < 0.05 was considered statistically significant and “n” represents the number of independent experiments conducted on distinct cell populations. 

## 3. Results

### 3.1. PI3K and mTOR Pathways are Involved in ATP-induced DNA Synthesis in VVECs

**Figure 1:**
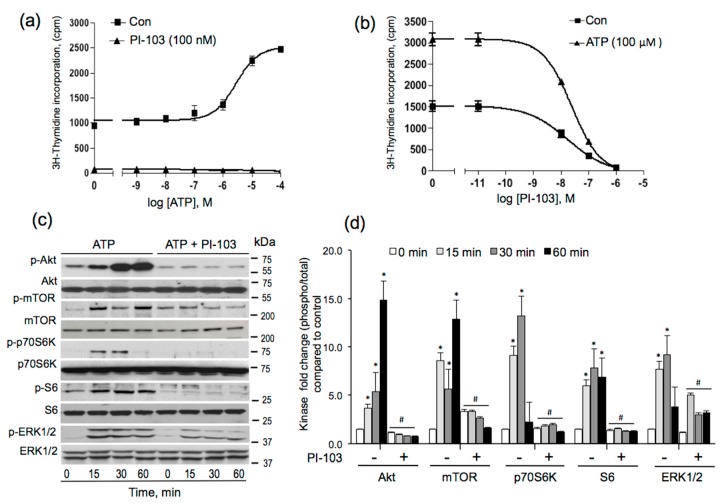
PI-103 inhibits ATP-stimulated DNA synthesis and mitogenic pathways in VVECs. Growth-arrested VVECs remained untreated or were pre-incubated with PI-103 (100 nM, 60 min) and stimulated with ATP (100 μM) in the presence of 0.125 μCi of [^3^H] thymidine for 24 h. Incorporated radioactivity was measured as described in the Materials and Methods Section **(a)** Effect of PI-103 (100 nM) on ATP-dependent increase in [^3^H] thymidine incorporation. **(b)** Concentration-dependent effect of PI-103 on ATP-stimulated [^3^H] thymidine incorporation; data on panels a and b represent the mean ± SEM from three to six independent experiments (n = 3–6), conducted on three distinct VVEC populations; **panel a:** *p* < 0.05 con vs. PI-103-treated; **panel b:** *p* < 0.05 con vs. ATP-treated (10^−11^–10^−8^ M) (one-way analysis of variance); panel b shows non-linear regression analysis of the dose response curves for PI-103; R^2^ = 0.99; IC_50 =_= 23 nM. **(c)** Western blot analysis of ATP-stimulated phosphorylation of intracellular kinases and S6 protein in the absence and the presence of PI-103 (100 nM). **(d)** Graphical presentation of the Western blot data; data on **panel d** represent the mean ± SEM from three independent experiments (n = 3), conducted on three distinct VVEC populations; ATP-stimulated phospho-protein levels at 15, 30, and 60 min were compared to time 0. The effect of PI-103 was determined individually at each time point in the control and ATP-treated cells; *p < 0.05 con vs. ATP-stimulated, #*p* < 0.05 PI-103-untreated vs. PI-103-treated for each kinase (unpaired, two-tailed *t* test).

Our previous studies established the role of PI3K and Rho-ROCK pathways in ATP release and ATP-mediated proliferative responses in VVECs [18]. To further investigate the role of these pathways in VVEC angiogenesis, we used PI-103, a combined pharmacological inhibitor of PI3K and mTOR [34,35]. The treatment of growth-arrested VVECs (72 h, DMEM without serum) with extracellular ATP (10^−10^–10^−4^ M) increased DNA synthesis in a concentration-dependent manner with a maximal effect observed at 10^−5^–10^−4^ M (Figure 1a). Pre-incubation with PI-103 (10^−12^–10^−6^ M) completely abolished basal and ATP-stimulated DNA synthesis (Figure 1b). We also verified the inhibitory effect of PI-103 on the phosphorylation of the intracellular kinases involved in cell growth and proliferation. As shown in Figure 1 (Panels c and d), stimulation with extracellular ATP (100 μM) increased the phosphorylation of Akt, mTOR, S6, and ERK1/2 at 15, 30, and 60 min, and p70S6K at 15 and 30 min. Treatment with PI-103 significantly attenuated ATP-induced phosphorylation of Akt, p70S6K, and S6, consistent with the involvement of PI3K-mTOR pathways in ATP-induced VVEC mitogenesis. In addition, PI-103 attenuated phosphorylation of ERK1/2, suggested a potential signaling cross-talk between PI3K and ERK pathways.

### 3.2. ATP-stimulated VVEC Migration and Tube Formation are PI3K- and mTOR-dependent

**Figure 2:**
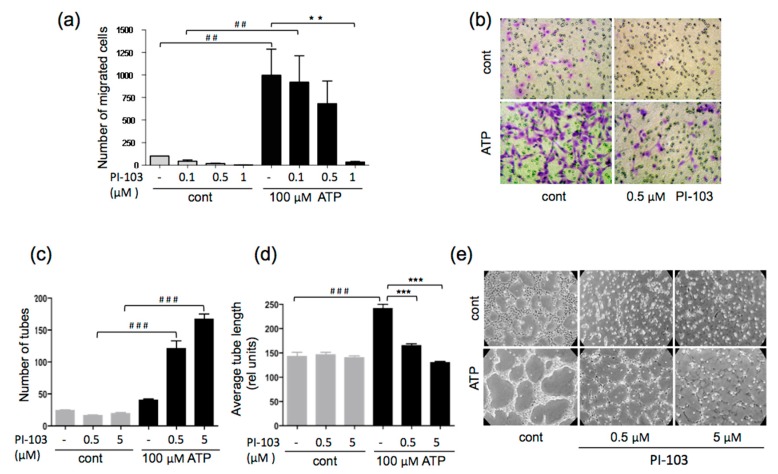
PI-103 inhibits ATP-stimulated migration and tube formation in VVEC. **(a)** Growth-arrested VVECs were seeded in Boyden chambers and remained untreated or were pretreated with PI-103 for 1 h, followed by adding ATP to the lower transwell compartment to initiate migration (100 μM). Migrated cells were counted at the end of the experiment, after 24 h. Shown are the data from four independent experiments (n = 4) conducted on four distinct VVEC populations; values are means ± SEM; ##*p* < 0.01, ATP-untreated vs. ATP-treated; **p < 0.01, PI-103-untreated vs. PI-103-treated. **(b)** Representative membrane filters with migrated cells, stained with crystal violet. **(c, d)** VVEC were plated on Growth Factor Reduced Matrigel as described in the Materials and Methods Section. Cells were untreated or pretreated with PI-103 (0.5 and 5 μM) for 1 h and stimulated with ATP (100 μM). Tube formation was assessed after 8 h incubation. Shown are number of tubes and average tube length from five independent experiments (n = 5) performed on three distinct VVEC populations; values are means ± SEM; ###*p* < 0.001 ATP-untreated vs. ATP -treated; ***p < 0.05 PI-103-untreated vs. PI-103-treated. **(e)** VVEC tube formation images from a representative experiment; *p* < 0.05 was considered significant (Panels a, c, d: one-way analysis of variance followed by Bonferroni post-test).

In addition to proliferation, endothelial migration and tube formation were important steps in the angiogenic process. The effect of PI-103 on the ATP-stimulated migration of VVECs was assessed using a modified Boyden chamber assay. PI-103 (0.1, 0.5, and 1 μM), in a concentration-dependent manner, decreased VVEC migration, with the maximal inhibition occurring at 1 μM (Figure 2a,b). Further, using a Matrigel assay, we examined the effect of PI-103 on ATP-induced tube formation.

As shown in Figure 2 (Panels c–e), PI-103 (0.5 μM and 5 μM) decreased the tube length and increased the number of tubes leading to a network of multiple poorly developed tube-like structures, confirming the role of the PI3K-mTOR pathway in ATP-induced VVEC morphogenetic response.

### 3.3. Transcription Factor Activation Profile Identifies Extracellular ATP-regulated TFs 

**Figure 3:**
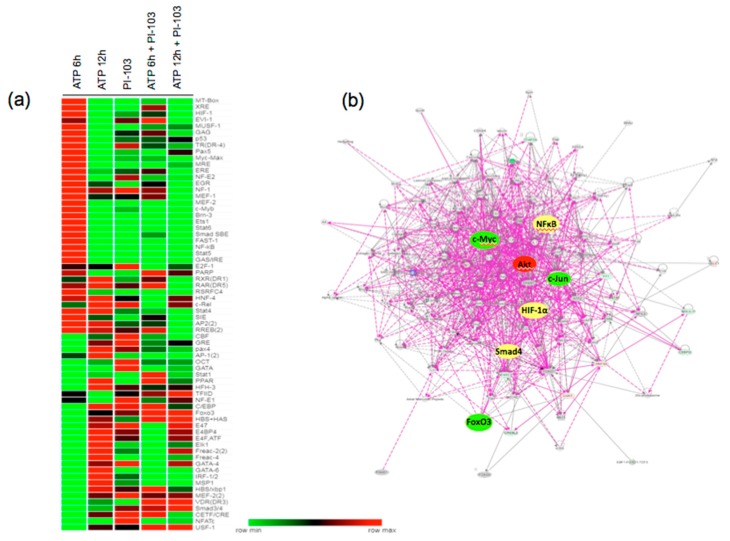
Effect of PI-103 on subsets of ATP-regulated transcription factors in VVEC. **(a)** Nuclear fractions of ATP- and PI-103- treated VVEC from 3 distinct populations were analyzed for DNA binding activity using TranSignal protein/DNA array. Heatmap analysis of the TFs that were significantly (p ≤ 0.05) altered in response to ATP and PI-103 (Morpheus software). The range for minimal and maximal fold change was automatically generated for each TF. **(b)** Network of ATP-activated genes obtained by Ingenuity pathway analysis.

Considering the involvement of PI3K-mTOR and ROCK pathways in the transcriptional control of gene expression, we examined the effect of PI-103 on ATP-induced TF activation, using TranSignal protein/DNA array. The TFs that showed more than 2-fold transactivation and inhibition in response to PI-103 treatment are represented in the heatmap generated using Morpheus software (Figure 3a). The data showed that treatment with extracellular ATP (100 μM, 6 h) resulted in the activation of about a half of TFs presented in the array (30 of 68), most of which are involved in the control of cell proliferation, growth, and transformation. Under the same conditions, however, ATP decreased DNA binding activity of another half of TFs (28 of 68). Stimulation with extracellular ATP for 12 h resulted in the activation of a subset of an additional 18 TFs, distinct from those activated after 6 h of ATP stimulation. PI-103 treatment significantly reduced the DNA-binding activity of TFs activated in response to ATP at 6 and 12 h. However, some TFs were activated in response to PI-103, which could represent TFs repressed by PI3K and mTOR pathways. Ingenuity pathway analysis demonstrated PI3K-mTOR-regulated multiple TFs with an interconnected network in ATP-stimulated VVECs, indicating a coordinate transactivation of various TFs that may be associated with the purinergic angiogenic response (Figure 3b). The pathway analysis revealed that c-Jun-, Foxo3a-, and c-Myc-formed nodes connecting several signaling networks associated with cell cycle, extracellular matrix, adhesion, cell junction, survival, and markers of endothelial phenotype, indicating the critical role of these TFs in ATP-mediated angiogenesis.

### 3.4. ATP-mediated Phosphorylation of c-Jun, Foxo3a, and c-Myc is PI-103-dependent

**Figure 4:**
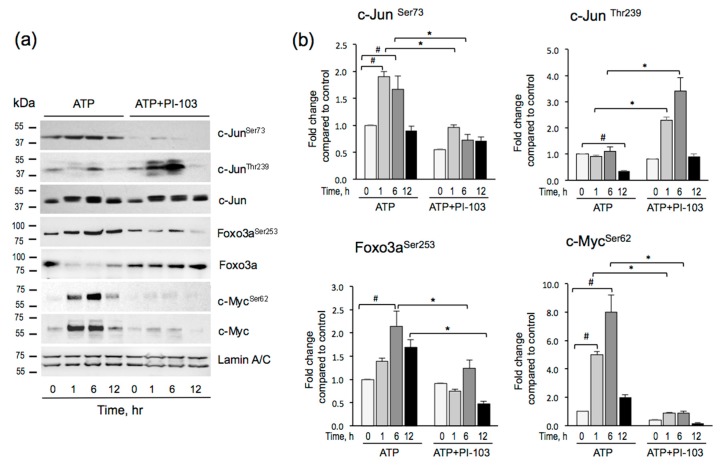
Effects of PI-103 on extracellular ATP-mediated activation of c-Jun, Foxo3a, and c-Myc in VVECs. Growth arrested VVECs, untreated or treated with PI-103 (100 nM, 1 h) were stimulated with ATP (100 μM) for 1, 6, and 12 h. **(a)** Representative Western blot analysis of phospho- and total c-Jun, Foxo3a, c-Myc in nuclear fractions. **(b)** Quantitative analysis of phospho-c-Jun^Ser73^, phospho-Foxo3a^Ser253^, and phospho-c-Myc^Ser62^ expression in nuclear fractions. Data show phosphorylated level of each TF normalized to Lamin A/C, a nuclear fraction marker. ATP-stimulated phospho-TF levels at 1, 6, and 12 h were compared to time 0. The effect of PI-103 was determined individually at each indicated time point in control and ATP-treated cells; the data represent the mean ± SEM from three to four independent experiments (n = 3–4) conducted on three cell populations. #*p* < 0.05 for comparison between cont vs. ATP; *p < 0.05 for comparison between control vs. PI-103 treatment (unpaired, two-tailed *t* test).

Based on the predicted essential roles of c-Jun, Foxo3a, and c-Myc in ATP-stimulated angiogenesis, we chose to determine the function of these TFs in more detail. The activity of c-Jun, Foxo3a, and c-Myc is regulated by several mechanisms, including upregulation, phosphorylation, and nuclear translocation [36,37]. A time course of c-Jun, c-Myc, and Foxo3a activation was obtained by measuring nuclear levels of total and phosphorylated forms of these TFs at different time points (1–12 h) in response to ATP (100 μM), with and without PI-103 (100 nM) treatments (Figure 4 a,b).

A time course of ATP-stimulated phosphorylation c-Jun, c-Myc, and Foxo3a showed a consistent increase in the phospho-levels of these proteins in the nuclear fraction. Phospho-c-Jun^Ser73^ and phospho-c-Myc^Ser62^ were increased at 1 and 6 h, whereas phospho-Foxo3a^Ser253^ peaked at 6 h and stayed elevated at 12 h. Pre-treatment with PI-103 (100 nM, 1 h) decreased phospho-c-Jun^Ser73^, phospho-Foxo3a^Ser253^, and phospho-c-Myc^Ser62^ levels. As GSK3β phosphorylates c-Jun on Thr^239^, repressing its DNA binding activity [38], we also determined the levels of phospho-c-Jun^Thr239^. Basal and ATP-stimulated phospho-c-Jun^Thr239^ were low, but ATP-stimulated phospho-c-Jun^Thr239^ significantly increased in PI-103 treated cells, consistent with the attenuation of Akt-mediated inhibitory phosphorylation of GSK3β. Therefore, these data showed that in VVECs, ATP-induced transactivation of c-Jun occurred by PI3K-Akt-mTOR pathway by increased phosphorylation at Ser73 and decreased phosphorylation at Thr^239^, the activation, and the inhibitory sites, respectively [39,40,41,42]. ATP mediated activation and PI-103 treatment had opposite effects on c-Jun^Thr239^ phosphorylation. The results confirmed that Akt inhibited GSK3β phosphorylation of c-Jun^Thr239^, thereby relieving tonic inhibition of the factor. Notably, some constitutive c-Jun was detected in the nuclear fraction, and it was slightly increased by ATP treatment (about 1.5 fold). In contrast, non-phosphorylated c-Myc exhibited nuclear localization patterns similar to c-Myc^Ser62^, suggesting that c-Myc phosphorylation is necessary for nuclear translocation. Foxo3a was present in the nuclear fraction under basal conditions, and ATP induced phosphorylation of Foxo3a by 2-fold. Phosphorylation of Foxo3a caused a decrease of total Foxo3a in the nuclear fraction which is consistent with inactivation by Akt phosphorylation and export to the cytoplasm. Treatment with PI-103 increased the levels of the unphosphorylated Foxo3a (the active form) in the nucleus.

### 3.5. Effect of siRNA to c-Jun, Foxo3a, and c-Myc on Angiogenic Responses in VVEC

**Figure 5:**
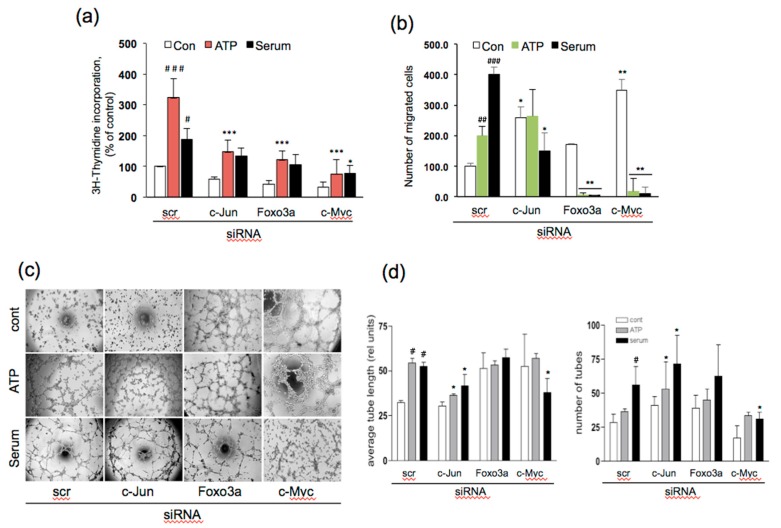
Effect of siRNA-mediated knockdown of c-Jun, Foxo3a, and c-Myc on ATP-stimulated proliferation, migration, and tube formation of VVECs. Cells were transfected with scramble siRNA or siRNA to c-Jun, Foxo3 and c-Myc using Dharmafect Reagent® following the protocol suggested by the manufacturer (Dharmacon). Cells were stimulated with ATP (100 μM) or serum (1% FBS), and proliferation, migration, and tube formation assays were conducted as described in the Materials and Methods Section. (**a**) [^3^H] thymidine incorporation-based DNA synthesis assay; (**b**) Boyden chamber migration assay; and (**c**-**d**) Matrigel tube formation assay. The data on all panels represent the mean ± SEM from six independent experiments (n = 6) performed on four VVEC populations; #*p* < 0.05, ##*p* < 0.01, ###*p* < 0.001 for comparison between cont vs. ATP- and serum-stimulated cells; *p < 0.05 for comparison between scramble siRNA-treated cells vs. TF-specific siRNA-treated cells for non-stimulated (cont), ATP-, or serum-stimulated conditions; (Panels a, b, and d: one-way analysis of variance followed by Bonferroni post-test);.

**Figure A1:**
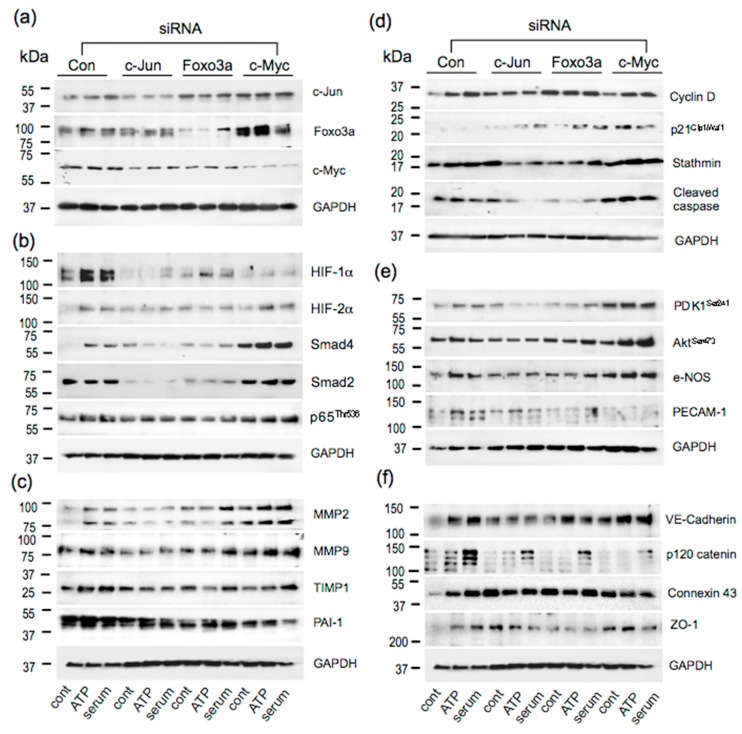
Effect of c-Jun, Foxo3a, and c-Myc KD on the expression of angiogenesis associated proteins. VVECs were transfected with scrambled siRNA or siRNA to c-Jun, Foxo3a, and c-Myc siRNA. Cells were stimulated with ATP (100 μM, 24 h), serum (1% FBS in DMEM, 24 h) or remained untreated. Total cell lysates were subjected to Western blot analysis. Shown are representative data from three to four (n = 3–4) independent experiments on three cell populations.

To assess the contribution of c-Jun, Foxo3a, and c-Myc in ATP- induced VVEC angiogenic responses, we examined the effect of siRNA-mediated knockdown (KD) of these TFs on proliferation, migration, and tube formation. VVECs treated with serum (1% FBS) were used for comparison to ATP responses. Western blot analysis showed 70–80% of the average decrease in c-Jun, Foxo3a, and c-Myc by respective siRNA 48 h post-transfection (Appendix, Figure A1). The KD of c-Jun, Foxo3a, and c-Myc significantly attenuated ATP-stimulated and, to some extent, serum-stimulated VVEC proliferation. The response to the serum was significantly decreased only by KD of c-Myc (Figure 5a). In the Boyden chamber assay, the KD of Foxo3a and c-Myc decreased ATP- and serum-induced migration (Figure 5b). The KD of c-Jun attenuated serum-induced, but not ATP-induced migration. Foxo3a and c-Myc KD increased basal VVEC migration, suggesting that under serum-starved conditions the presence of these proteins may be inhibitory to angiogenesis.

In the Matrigel angiogenic assay, ATP- and serum-induced tube formation (average tube length) was inhibited by c-Jun KD, not by Foxo3a KD. C-Myc KD inhibited serum- but not ATP-induced tube formation. In addition, Foxo3a and c-Myc KD evidently increased tube formation under basal conditions. However, the effect did not reach statistical significance (Figure 5 c,d). Notably, c-Jun KD-mediated decreases in ATP- and serum-induced tube length were accompanied by an increased number of shorter tubes. This tendency was also observed in Foxo3a KD cells (Figure 5 c,d). Taken together, these data demonstrate that c-Jun is required for ATP- and serum-induced proliferation and tube formation and serum-induced migration. Foxo3a and c-Myc are required for ATP- and serum-induced proliferation and migration. Thus, c-Jun, Foxo3a, and c-Myc have distinct roles in angiogenic responses in VVECs, and their activation is stimulus-specific.

### 3.6. Effects of c-Jun, Foxo3a, and c-Myc KD on the Expression of Angiogenesis Associated Proteins 

**Figure 6:**
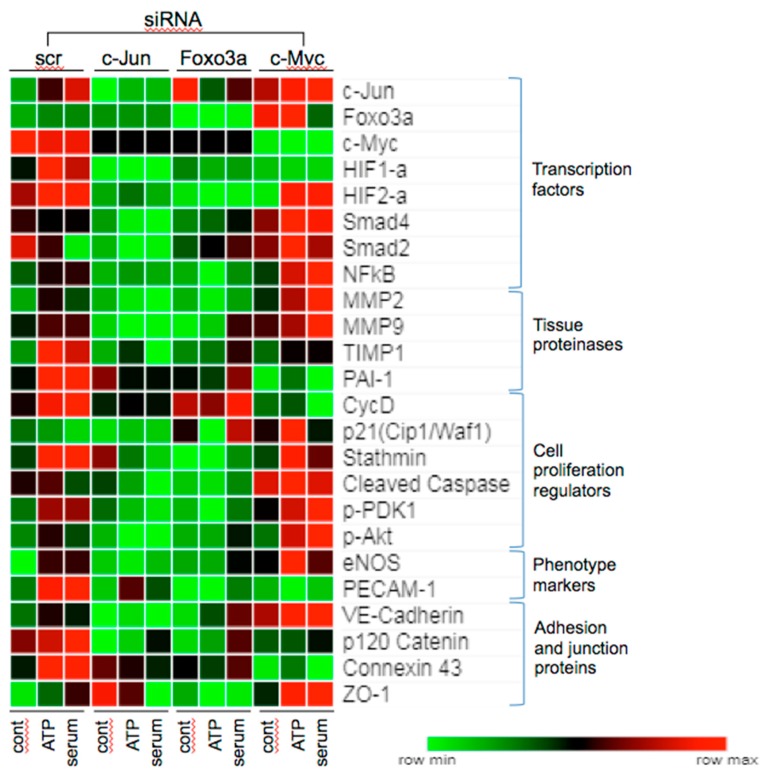
Effect of c-Jun, Foxo3a, and c-Myc siRNA on the expression of select angiogenesis associated proteins. VVECs were transfected with scrambled siRNA or siRNA to c-Jun, Foxo3, and c-Myc as described in the Materials and Methods Section. Cells were stimulated with ATP (100 μM, 24 h) or serum (FBS, 1% in DMEM, 24 h), and total lysates were subjected to Western blot (WB). Heatmap (Morpheus Analysis Software) was plotted based on densitometric analysis of WB data (normalized to non-stimulated scramble siRNA-transfected cells). By default, values in the heat map are mapped to colors using a relative color scheme using the minimum and maximum of each row independently. Each square on the heatmap represents a protein of interest normalized to GAPDH (data represent average from three experiments (n = 3) conducted on three cell populations).

Angiogenesis is a coordinated multistep process, in which endothelial cells dynamically alter gene expression involved in neovessel formation and endothelial maturation [43,44]. To further understand the role of c-Jun, c-Myc, and Foxo3a in VVEC angiogenesis, we analyzed the effect of KD of these TFs on the expression of select angiogenesis-associated target proteins. Since these TFs formed overlapping functional and signaling networks (Figure 3b), we first examined whether there was a cross-regulation among these TFs. Our results showed that KD of c-Jun decreased c-Myc expression, KD of c-Myc increased c-Jun and Foxo3a expression, and KD of Foxo3a significantly increased c-Jun expression under basal conditions (Figure 6), suggesting a possible functional and regulatory cross-talk between these proteins in angiogenesis. Based on pathway analysis data (Figure 3b), we also examined ATP-induced levels of HIF-1α, HIF-2α, NFκB (p65), Smad2, and Smad4, known to have pro-angiogenic and pro-inflammatory roles in endothelial cells. KD or c-Jun significantly decreased levels of HIF-1α, HIF-2α, Smad2, and Smad4 in ATP- and serum-stimulated cells. Foxo3a KD decreased basal, ATP- and serum-stimulated levels of HIF-1α, HIF-2α, and NFκB (p65). C-Myc KD decreased basal, ATP- and serum-stimulated HIF-1α, but increased ATP- and serum-stimulated Smad4, ATP-stimulated Smad2, and serum-stimulated NFκB (p65) levels (Figure 6). These results indicate that c-Jun, Foxo3a, and c-Myc regulated the angiogenic effects of ATP and serum by modulating the levels of multiple pro-inflammatory and pro-angiogenic TFs, which indicates that c-Jun, Foxo3a, and c-Myc regulate the angiogenic effects of ATP and serum by modulating levels of multiple pro-inflammatory and pro-angiogenic TFs.

Tissue remodeling and degradation of the vascular wall basement membrane, which allows endothelial cells to migrate and invade the surrounding tissue, requires pro-angiogenic metalloproteinases (MMPs), MMP2, MMP9 and their regulators, TIMP1 and PAI-1 [45]. KD of c-Jun inhibited the ATP- and serum-induced expression of proangiogenic MMP2, MMP9, TIMP1, and PAI-1 (Figure 6). KD of Foxo3a decreased ATP- and serum-induced MMP2, TIMP1, and PAI-1; and ATP-induced MMP9 levels. In contrast, KD of c-Myc increased serum-induced MMP 2 and MMP 9 expression; decreased serum-induced TIMP1; and decreased basal, ATP-, and serum-stimulated PAI-1 levels. These results suggest that c-Jun and Foxo3a are required for ATP-induced MMP expression in VVECs, while c-Myc is inhibitory to expression. They also suggest c-Jun upregulated, and c-Myc repressed the expression of tissue remodeling genes under all conditions, while Foxo3a repressed only serum-induced genes.

Next, we determined the effect of KD of c-Jun, Foxo3a, and c-Myc on the levels of select proteins implicated in proliferation and apoptosis—the responses important in angiogenesis. KD of c-Jun decreased ATP- and serum-induced cyclin D, stathmin, and cleaved caspase 3 levels. Foxo3a KD decreased basal, ATP-, and serum-induced stathmin and cleaved caspase 3 levels, had little effect on serum-induced cyclin D, and increased basal- and serum-stimulated p21^Cip1/Waf1^ levels. C-Myc decreased ATP- and serum-induced cyclin D and serum-induced stathmin while it increased the ATP- and serum-induced expression of anti-proliferative p21^Cip1/Waf1^ and apoptotic cleaved caspase 3 levels (Figure 6). These results indicate that c-Jun and c-Myc may control proliferation by increasing cyclin D levels, while Foxa3a suppresses proliferation by increasing p21^Cip1/Waf1^ levels. The increases in p21^Cip1/Waf1^ levels by c-Myc KD may be due to upregulation of Foxo3a or Smad. The increase in levels of cleaved caspase3 by c-Myc KD suggests that it may promote cell survival.

PDK and Akt activation are involved in maintaining the eNOS function and survival of endothelial cells [26]. KD of c-Jun and Foxo3a decreased, but KD of c-Myc increased phospho-PDK and phospho-Akt levels. KD of c-Jun also decreased TIMP-1, which functions as a positive regulator of PDK [46]. The decrease in TIMP-1 by c-Jun KD also correlated with reduced phospho-PDK1, phospho-Akt^Ser473^ and eNOS levels (Figure 6). Foxo3a KD decreased basal and ATP-induced phospho-PDK1, phospho-Akt^Ser473^ and eNOS levels, whereas c-Myc KD increased serum-stimulated levels of PDK1, phospho-Akt^Ser473^, and ATP-stimulated eNOS, suggesting differential regulation of these target proteins by TFs and angiogenic stimuli. The endothelial marker PECAM-1 was reduced by Foxo3a and c-Myc KD (Figure 6), indicating a role for these TFs in VVEC endothelial differentiation and activation. 

Assembly of endothelial adherens and tight junctions is essential to vascular homeostasis, permeability, and angiogenesis [47]. Tight junction proteins suppress proliferation to allow endothelial differentiation by remodeling adherens junctions and adhesion to the extracellular matrix. The increase in endothelial connexin 43 expression and decrease in VE-cadherin increased microvessel permeability, and was involved in angiogenic tube formation and in cell migration [48]. In VVECs, KD of c-Jun decreased basal, ATP-, and serum-stimulated VE-cadherin, and p120 catenin expression; Foxo3a decreased basal and serum-induced levels of VE-cadherin, and p120 catenin KD of c-Myc increased ATP-and serum-induced VE-cadherin (Figure 6). These results suggest that c-Myc inhibits barrier function in VVECs. Connexin 43 levels were decreased by siRNA to cMyc>Foxo3a>c-Jun. ZO-1 regulated actomyosin organization and had a role in cell migration and barrier formation in angiogenesis [49]. ZO-1 expression was increased by c-Myc siRNA in basal and stimulated conditions. Foxo3a siRNA decreased ZO-1 expression even under basal conditions, while c-Jun siRNA increased ZO-1 under basal conditions. Together, our data demonstrate that in VVEC angiogenesis, tight junction and adhesion proteins are differentially regulated by a combination of these TFs. C-Jun and c-Myc appear to have a role in responses to angiogenic stimuli, and Foxo3a in endothelial homeostasis.

## 4. Discussion

Preclinical and clinical studies demonstrate that pathologic angiogenesis of the VV plays a critical role in the development of cardiovascular diseases, including atherosclerosis and PH [1,2,3,4,5,6,7]. Angiogenesis involves multiple steps including degradation of the extracellular matrix, endothelial cell migration, proliferation, tube formation, and phenotypic differentiation. Various signaling pathways contribute to these processes, finally converging on TFs. However, prior to this study, the transcriptional regulation of VV angiogenesis and the underlying purinergic signaling mechanisms had not been examined. We identified a network of ATP-sensitive TFs and found that at least 68 are regulated by PI3K-mTOR kinase pathways, and known to play an established role in autocrine/paracrine regulation of ATP-mediated VVEC angiogenesis. Our findings also revealed that c-Jun, Foxo3a, and c-Myc are ATP-inducible PI3K-Akt-mTOR-regulated TFs that are consistently activated in most ATP-stimulated VVEC populations, and form the nodes of overlapping signaling networks. These observations led us to focus on c-Jun, Foxo3a, and c-Myc as potential fundamental regulators of ATP-driven VVEC angiogenesis. 

TFs are critical for the regulation of vessel formation, growth, and development [50,51,52,53]. Both transcriptional activation and repression are used to control angiogenic pathways. Dynamic changes in gene expression during angiogenic processes are achieved by activation of transcription factors whose activities are modified by post-transcriptional modifications. Approximately 2000 TFs comprise about 8% of the genome, and many of them are regulated by the PI3K-Akt-mTOR signaling axis [34]. In VVECs, c-Jun and c-Myc are activated by Akt, whereas Foxo is inhibited by [21,22,36,54,55,56,57,58]. Increasing evidence suggests that interaction between TFs in endothelial cells play a crucial role in vascular network formation and maintenance of vascular integrity [56]. 

**Figure 7:**
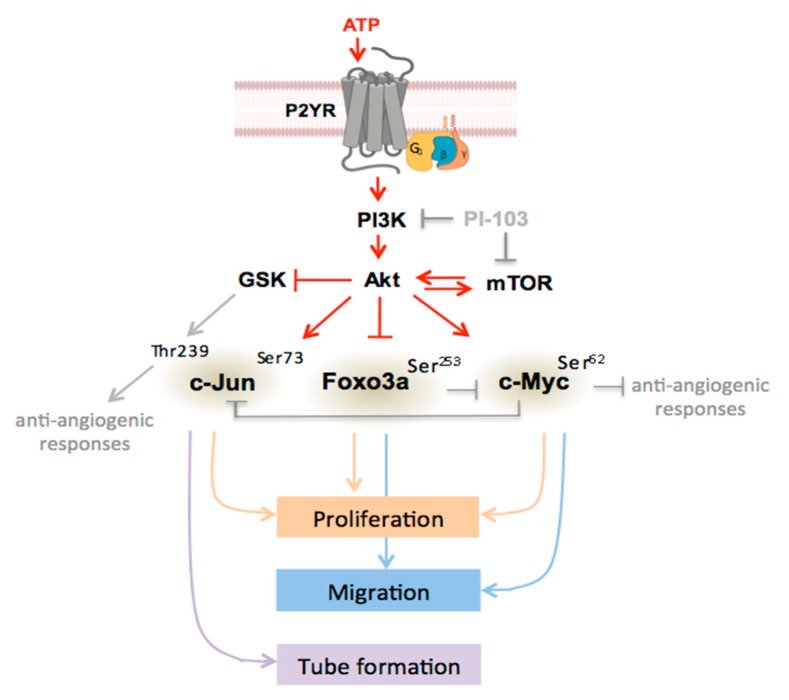
Schematic illustration of ATP-mediated regulation of angiogenic responses in VVEC. Extracellular ATP stimulates purinergic receptor (P2YR)—mediated activation of intracellular PI3K, Akt, mTOR pathways leading to the activation of transcription factors c-Jun, Foxo3a, and c-Myc that have been identified as predominant transcriptional regulators of ATP-mediated angiogenesis in VVECs. Activation of these TFs results in the regulation of functional angiogenic responses—proliferation, migration, and tube formation. In addition, shown here are a potential anti-angiogenic contribution of GSK-mediated c-Jun phosphorylation at Thr239 and a potential angiogenic contribution of c-Myc via the suppression of anti-angiogenic genes.

Under hypoxic conditions, elevated tissue levels of multiple pro-angiogenic growth factors and extracellular nucleotides can stimulate angiogenesis [18,59,60,61,62]. Although both G protein-coupled and tyrosine kinase receptors are known to activate growth-promoting PI3K/Akt/mTOR signaling, the TF activation mediated by purinergic receptors remains poorly investigated. In our study, the angiogenic effects of ATP were compared with serum and used as a source of pro-angiogenic growth factors. The observations suggest that different combination of TFs are involved in purinergic and growth factor-mediated angiogenic regulation, for example, in the activation c-Jun, Foxo3a, and c-Myc required for ATP-induced proliferation compared to c-Myc for serum-induced proliferation. All three TFs are involved in serum-induced migration compared to Foxo3a and c-Myc for ATP-induced responses (Figure 5). In addition, the differential regulation of tube formation was also observed. Considering that extracellular ATP may act in an additive manner with growth factors to stimulate vascular cell proliferation [24,63], our data may suggest that purinergic angiogenic responses in the hypoxic and inflammatory microenvironment can be amplified or modulated by endogenous growth factors to regulate VV expansion. The ATP-regulated angiogenic responses are illustrated in Figure 7. 

The link between the activation of specific TFs and the genes regulating different angiogenic responses and vascular cell phenotypes is not fully elucidated and remains the subject of intensive investigation [31,64]. In VVEC manipulation of c-Jun and c-Myc (early response transcription factors) by siRNA demonstrated that they control the activation of hypoxia-sensitive factors, including HIF-1α, HIF-2α, NFκB, and Smads, suggesting a functional cross-talk between angiogenesis-associated TFs in hypoxic and inflammatory conditions. Our data indicate that c-Jun predominantly regulates pro-angiogenic genes in VVECs. Previous studies showed that c-Jun expression positively correlates with the angiogenesis of normal and tumor blood vessels, and targeting c-Jun inhibits vascular permeability and inflammation [50,65,66,67]. siRNA to c-Jun, c-Myc and, to a lesser extent, Foxo3a KD decreased levels of HIF-1α, suggesting the role of these TFs in HIF-1α stabilization under pro-angiogenic conditions in normoxia. Association of c-Jun with HIF-1α via its oxygen-dependent degradation (ODD) domain potentiates its transcriptional activity by preventing proteasome degradation. In addition, co-operation of HIF-1α and c-Jun increases VEGF and Il-8 expression and promotes angiogenesis [50]. The potential co-regulation and co-expression of ATP- and hypoxia- sensitive TFs may contribute to hypoxia-induced VV angiogenesis, which is associated with adventitial inflammation, structural vascular remodeling and metabolic adaptations [3]. 

c-Myc functions as a master regulator of various genes involved in cell growth and transformation during angiogenesis, vascular and hematopoietic development [57,68,69]. c-Myc promotes pathogenic effects of IL-6 in PH by decreasing TGFβ and pro-apoptotic factors, but up-regulating pro-survival factors [70]. Interaction between c-Myc and Smad pathways appears to be important in the regulation of angiogenesis. Even though c-Myc is downstream of the Smad pathway, its interaction with Smads is reported to inhibit the anti-angiogenic effects of TGFβ [71]. Our results showed that Smads and NFκB were suppressed by c-Myc. Inactivating c-Myc clearly enhanced signaling through Smads and increased the expression of TGFβ-regulated genes like p21Cip/Waf, NFkB, MMP2, MMP9, and eNOS. It is possible that in VVECs c-Myc may antagonize the anti-angiogenic role of TGFβ. Loss of TGFβ signaling and Smad levels was described in the monocrotaline model of rat PAH which involves endothelial injury [72]. In addition, c-Myc knockdown in human endothelial cells was shown to promote a senescent phenotype associated with inflammation [73]. In VVECs, c-Myc KD increased p21^Cip/Waf1^ and NFkB—markers of a senescent phenotype [73,74]. The senescent state of VVECs is likely to contribute to decreased ATP-induced proliferation and migration and decreased expression of cyclin D with siRNA. 

c-Myc can both activate and repress transcription, and other studies have shown a significant overlap between the group of genes repressed by Foxo proteins and activated by c-Myc [75,76]. The phosphorylation of Foxo TFs by Akt alleviates the inhibition of c-Myc target genes by Foxo [54]. The Foxo3a mutant cannot be phosphorylated and inactivated by Akt to repress the multiple target genes of c-Myc that are involved in cell proliferation and block c-Myc-dependent proliferation [77]. Temporal differences in activated Akt can therefore dynamically alter proliferative state by inducing cyclin D levels and c-Myc or by decreasing p21^Cip^ levels by inactivating Foxo3a. Stathmin controls cellular proliferation by promoting microtubule depolymerization and is regulated by c-Jun and Foxo3a in VVECs, suggesting multiple mechanisms for control of proliferation. 

Foxo family TFs play a key role in various physiological responses, including angiogenesis and postnatal neovascularization [53]. Importantly, c-Jun and Foxo1 pathways promote pulmonary artery endothelial cell growth and angiogenesis during PAH [78]. Previous studies demonstrated that Foxo1 and Foxo3a specifically regulate a non-redundant, but overlapping set of angiogenic and vascular remodeling genes and are the predominant Foxo subtypes in endothelial cells [28,79]. In VVECs, Foxo3a KD decreases the expression of multiple angiogenesis-associated proteins, showing a similar, but not identical, profile of targets regulated by c-Jun. Consistent with the described mechanisms of Foxo regulation [23], we showed Akt-mediated phosphorylation of Foxo3a at Ser253 and its export from the nucleus. However, KD of Foxo3a decreased proliferation and migration of VVECs in response to ATP, suggesting Foxo1 may have a compensatory role in VVEC angiogenesis. 

The activation of the PI3K-Akt signaling pathway resulted in the phosphorylation of several Akt substrates that are also important in controlling the balance between cell survival and apoptosis [80]. For example, PAI-1 -/- KO endothelial cells showed increased Akt^Ser473^ levels and proliferation [81]. Increased Akt^Ser473^ and decreased PAI-1 levels in c-Myc KD VVECs may indicate that, in addition to its fibrinolytic activity, PAI is also involved in angiogenic regulation. In atherogenic LDLR^−/−^/ApoB48-deficient mice, stimulation of plasmin activity by truncated PAI-1 protein was antiangiogenic and caused regression and collapse of adventitial VV [82]. In VVECs, decreased migration observed with c-Myc and Foxo3a KD may suggest a role for PAI-1 in migration. c-Myc KD significantly increased levels of cleaved caspase 3 and, paradoxically, also increased the levels of p-PDK1, p-Akt, and eNOS—proteins associated with survival signals in endothelial cells—suggesting that cleaved caspase 3 may not be pro-apoptotic in VVECs. For example, non-apoptotic functions of caspase 3 have been reported in differentiating lens epithelium, keratinocytes, skeletal myocytes, and in activated T cells. It can also be observed in certain tumors and predicts better chances of survival [83,84,85].

Our results suggest that c-Myc may have a dual role in angiogenic regulation by functioning as a transcriptional activator and repressor, maintaining the angiogenesis balance in physiological states. C-Myc ^Ser62^ phosphorylated by Akt may have a role in initiating angiogenesis. However, when Akt activity is low, c-Myc can be phosphorylated at Thr58 by GSK, which targets it for rapid proteolysis by the ubiquitin pathway, allowing the expression of c-Myc genes controlling vessel stabilization [86]. Akt, therefore, may play an important role in determining the role of c-Myc in regulating angiogenesis. 

Transcriptional control of VV expansion and its contribution to pathological vascular remodeling remains important, yet also an area not fully explored. It should be noted that VVEC stimulation with ATP and serum resulted in expression changes of both overlapping and specific angiogenesis-associated protein profiles regulated by c-Jun, Foxo3a, and c-Myc TFs. Again, these observations support the idea that purinergic regulation of VV angiogenesis in the hypoxic and inflammatory microenvironment is likely integrated into the regulatory circuits of multiple growth factors and other endogenous angiogenic regulators. The evidence of the involvement of c-Jun, Foxo3a, and c-Myc in ATP-regulated VV angiogenesis is in line with the previously reported role of these TFs in proliferative, survival, and inflammatory responses in the pulmonary vasculature in PH [70,78]. Importantly, ATP affects all the steps of angiogenic processes including degradation of the extracellular matrix, cell proliferation, migration, tube formation, and changes in endothelial phenotype. Given that elevated extracellular ATP levels can be observed at the sites of vascular injury and inflammation and that purinergic receptors are druggable targets, our data may suggest that targeting P2YR purinergic receptors by selective high-affinity agonists, antagonists, and a limited number of critical TFs, could sufficiently reduce VV angiogenesis and, in turn, reduce pathologic vascular remodeling and perivascular inflammation in PH and other cardiovascular diseases.

## 5. Conclusions

In this study, we demonstrated that c-Jun, c-Myc, and Foxo3a TFs are key ATP-inducible PI3K-Akt-mTOR-regulated angiogenic TFs in PA VVECs. siRNA-mediated knockdown revealed specific involvement of these TFs in different steps of VVEC angiogenesis and identified select target proteins, including cell cycle regulators, adhesion and junctional proteins, tissue proteinases, and phenotype markers. Considering increasing evidence of the importance of the VV in the pathogenesis of cardiovascular diseases, a potential clinical significance of our study would be an application of novel vascular therapies by targeting purinergic receptors and downstream signaling pathways, associated with VV dysfunction and pathological vascular remodeling.

## Appendix A

**Table A1:** Sources of antibodies.

Antibody	Source	Catalog#	Dilution
p-Akt^Ser473^	CST	CST4060	1:1000
Akt	CST	CST9272	1:1000
p-S6	CST	CST2211	1:1000
S6	CST	CST2217	1:1000
p-ERK1/2	CST	CST4370	1:1000
ERK	CST	CST9102	1:1000
p-mTOR	CST	CST5536	1:1000
mTOR	CST	CST2972	1:1000
p-p70S6K^Thr421/Ser424^	CST	CST9204	1:1000
p70S6K	CST	CST9202	1:1000
p-PDK^Ser241^	CST	CST3061	1:1000
Lamin AC	CST	CST477	1:6000
c-Jun^Ser73^	CST	CST8752	1:1000
c-Jun^Thr239^	Genetex	GTX50103	1:1000
c-Jun	CST	CST9165	1:1000
FoxO3a^Ser253^	CST	CST13129	1:1000
FoxO3a	CST	CST12829	1:1000
Myc^Ser62^	CST	CST13748	1:1000
Myc	CST	CST9402	1:1000
HIF-1α	Novus	NB100654	1:1000
HIF-2	Novus	NB100132	1:1000
Smad2	CST	CST 5339	1:1000
Smad4	CST	CST4653	1:1000
p65^Thr536^	CST	CST4887	1:1000
MMP2	CST	CST4022	1:1000
TIMP1	CST	CST8946	1:1000
MMP9	CST	CST27647	1:1000
PAI-1	CST	CST11907	1:1000
Cyclin D1	SCBT	SC-753	1:1000
Stathmin	Abcam	CST3352	1:1000
PECAM-1	CST	NBP171663	1:1000
p21^Cip1/Waf1^	CST	CST2947	1:1000
Cleaved caspase3	CST	CST9664	1:500
PDK1	CST	CST3062	1:1000
e-NOS	CST	CST32027	1:1000
VE-Cadherin	CST	CST2158	1:1000
Connexin 43	CST	CST3512	1:1000
p120 Catenin	CST	CST4989	1:1000
ZO-1	CST	CST5406	1:1000
GAPDH	CST	CST2118	1:5000
